# Prognostic impact of urokinase-type plasminogen activator (uPA) and its inhibitor (PAI-1) in cytosols and pellet extracts derived from 892 breast cancer patients

**DOI:** 10.1038/sj.bjc.6690191

**Published:** 1999-03

**Authors:** J H de Witte, C G J Sweep, J G M Klijn, N Grebenschikov, H A Peters, M P Look, Th H van Tienoven, J J T M Heuvel, W L J van Putten, Th J Benraad, J A Foekens

**Affiliations:** Department of Chemical Endocrinology, University Hospital Nijmegen, Geert Grooteplein 8, PO Box 9101, 6500 HB Nijmegen, The Netherlands; Division of Endocrine Oncology (Department of Medical Oncology), Rotterdam Cancer Institute (Dr Daniel den Hoed Kliniek)/Academic Hospital Rotterdam, PO Box 5201, Rotterdam, 3008 AE, The Netherlands; Department of Statistics, Rotterdam Cancer Institute (Dr Daniel den Hoed Kliniek)/Academic Hospital Rotterdam, PO Box 5201, Rotterdam, 3008 AE, The Netherlands

**Keywords:** urokinase-type plasminogen activator, plasminogen activator inhibitor, enzyme-linked immunosorbent assay, cytosol, breast cancer, prognostic impact

## Abstract

To evaluate the clinical relevance of urokinase-type plasminogen activator (uPA) and its type-1 inhibitor (PAI-1) measured by a recently developed enzyme-linked immunosorbent assay (ELISA), we analysed both components in samples derived from 892 patients with primary breast cancer (median follow-up 99 months). The assays were performed in cytosolic extracts as well as in corresponding detergent extracts of pellets obtained after ultracentrifugation, which was carried out when preparing the cytosolic fractions for routine steroid hormone receptor determination. Statistically significant correlations were found between the cytosolic levels and those determined in the pellet extracts (Spearman correlation coefficient *r*_s_ = 0.60, *P* < 0.0001 for uPA and *r*_s_ = 0.65, *P* < 0.0001 for PAI-1). Furthermore, strong correlations were found between the levels of both uPA (*r*_s_ = 0.85, *P* < 0.0001) and PAI-1 (*r*_s_ = 0.90, *P* < 0.0001) in the cytosols and their levels previously measured with ELISAs based on commercial reagents. In both Cox univariate and multivariate analysis, high cytosolic levels of uPA or PAI-1 were significantly associated with increased rates of relapse and death. The levels of uPA and PAI-1 in the pellet extracts also provided prognostic information, although to a lesser extent compared with the cytosolic extracts. The prediction of prognosis on the basis of uPA and PAI-1 assessed by an alternative ELISA once again emphasizes the established prognostic role and usefulness of these parameters in selection of breast cancer patients at high or low risk of recurrence. © 1999 Cancer Research Campaign


*British Journal of Cancer* (1999) **79**(7/8), 1190–1198
© 1999 Cancer Research Campaign
Article no. bjoc.1998.0191

### Prognostic impact of urokinase-type plasminogen activator (uPA) and its inhibitor (PAI-1) in cytosols and pellet extracts derived from 892 breast cancer patients

**JH de Witte[1], CGJ Sweep[1], JGM Klijn[2], N Grebenschikov[1], HA Peters[2], MP Look[2], ThH van Tienoven[1], JJTM Heuvel[1], WLJ van Putten[3], ThJ Benraad[1] and JA Foekens[2]**

[1]Department of Chemical Endocrinology, University Hospital Nijmegen, Geert Grooteplein 8, PO Box 9101, 6500 HB Nijmegen, The Netherlands; [2]Division of Endocrine Oncology (Department of Medical Oncology); [3]Department of Statistics, Rotterdam Cancer Institute (Dr Daniel den Hoed Kliniek)/Academic Hospital Rotterdam, PO Box 5201, 3008 AE Rotterdam, The Netherlands

**Summary** To evaluate the clinical relevance of urokinase-type plasminogen activator (uPA) and its type-1 inhibitor (PAI-1) measured by a recently developed enzyme-linked immunosorbent assay (ELISA), we analysed both components in samples derived from 892 patients with primary breast cancer (median follow-up 99 months). The assays were performed in cytosolic extracts as well as in corresponding detergent extracts of pellets obtained after ultracentrifugation, which was carried out when preparing the cytosolic fractions for routine steroid hormone receptor determination. Statistically significant correlations were found between the cytosolic levels and those determined in the pellet extracts (Spearman correlation coefficient $r_s = 0.60$, $P < 0.0001$ for uPA and $r_s = 0.65$, $P < 0.0001$ for PAI-1). Furthermore, strong correlations were found between the levels of both uPA ($r_s = 0.85$, $P < 0.0001$) and PAI-1 ($r_s = 0.90$, $P < 0.0001$) in the cytosols and their levels previously measured with ELISAs based on commercial reagents. In both Cox univariate and multivariate analysis, high cytosolic levels of uPA or PAI-1 were significantly associated with increased rates of relapse and death. The levels of uPA and PAI-1 in the pellet extracts also provided prognostic information, although to a lesser extent compared with the cytosolic extracts. The prediction of prognosis on the basis of uPA and PAI-1 assessed by an alternative ELISA once again emphasizes the established prognostic role and usefulness of these parameters in selection of breast cancer patients at high or low risk of recurrence.

**Keywords:** urokinase-type plasminogen activator; plasminogen activator inhibitor; enzyme-linked immunosorbent assay; cytosol; breast cancer; prognostic impact

Proteolytic enzymes are involved in cancer invasion and metastasis by their capability to degrade basement membranes and extracellular matrix proteins surrounding normal tissue (Liotta et al, 1982; Danø et al, 1985; Mignatti and Rifkin, 1993; Andreasen et al, 1997). One of the key enzymes is urokinase-type plasminogen activator (uPA) that, in its active form, catalyses the conversion of plasminogen into plasmin, which has the ability to degrade several components of the extracellular matrix and to activate some prometalloproteases (Danø et al, 1985; Duffy, 1992; Mignatti and Rifkin, 1993; Andreasen et al, 1997). Binding of uPA to its receptor (uPAR) strongly enhances uPA-mediated plasminogen activation and localizes the proteolytic activities on the cell surface (Danø et al, 1994). The proteolytic activity of uPA is controlled by two members of the serpin family of protease inhibitors, i.e. plasminogen activator inhibitor type-1 (PAI-1) and type-2 (PAI-2) (Andreasen et al, 1990). By forming complexes with uPA bound to uPAR, PAI-1 promotes the clearance of proteolytic activities from the cell surfaces and recycling of unbound uPAR back to the cell surface, thereby regulating the overall invasive and metastatic behaviour of cancer cells (Andreasen et al, 1997; Chapman et al, 1997).

*Received 27 February 1998*
*Revised 23 June 1998*
*Accepted 3 August 1998*

*Correspondence to:* JH de Witte

Several investigations have demonstrated the prognostic value of uPA and PAI-1 in breast cancer patients, and high levels of both components have been shown to be independent and strong prognostic parameters predicting disease recurrence and overall survival (reviewed in Duffy, 1996; Schmitt et al, 1997). Mostly, ELISA methods have been used for determination of both uPA and PAI-1, either in detergent-treated tumour tissue extracts (Jänicke et al, 1989, 1990, 1991, 1993, 1994*a*) or in cytosolic extracts routinely prepared for the assessment of steroid hormone receptors [oestrogen receptor (ER) and progesterone receptor (PgR)] (Duffy et al, 1990; Foekens et al, 1992; Spyratos et al, 1992; Grøndahl-Hansen et al, 1993; Foekens et al, 1994; Jänicke et al, 1994*a*; Fernö et al, 1996). Recently, two new ELISAs were developed which proved to be suitable for the determination of uPA and PAI-1 in breast tumour tissue (Grebenschikov et al, 1997). These in-house ELISAs are based on a combination of polyclonal antibodies used in a sandwich assay format to which special features are attributed because uPA and PAI-1, and eventually complexes between both components, can be simultaneously assessed in one and the same ELISA frame. In the present study, the in-house ELISAs were applied to the determination of uPA and PAI-1 in breast tumour cytosols and in corresponding detergent extracts of pellets obtained after ultracentrifugation carried out when preparing the cytosolic fractions derived from 892 primary breast cancer patients. The prognostic impact of the uPA and PAI-1 antigen levels in cytosols and pellet extracts was subsequently evaluated to assess the established clinical significance of the two components. Furthermore, the levels of uPA and PAI-1 were correlated to those obtained earlier with ELISAs based on commercial reagents.

### MATERIALS AND METHODS

### Patients and tumour characteristics

In the present study, 892 patients with primary operable breast cancer (modified mastectomy, 446 patients; breast-conserving lumpectomy, 446 patients) were included. Inclusion criteria for patients from whom tumour biopsies or cytosol samples were stored in the tumour bank of the Rotterdam Cancer Centre (Dr Daniel den Hoed Kliniek) were: (i) primary diagnosis of breast cancer between 1979 and 1989; (ii) no signs of distant metastasis at diagnosis; (iii) no previous diagnosis of carcinoma, with the exception of basal cell skin cancer and cervical cancer stage Ia; (iv) no evidence of disease within 1 month after primary surgery. In cases of mastectomy after an initial lumpectomy for residual disease, the mastectomy is considered as (part of) primary treatment. The median number of lymph nodes removed surgically was 11. Patients without primary surgery or patients who received neoadjuvant treatment before primary surgery were excluded. Median age of the patients at the time of surgery was 56 years (range 25–89 years). Radiotherapy was given to 82% of the patients: on the breast/thoracic wall to 617 patients and/or on the axilla to 262 patients, and/or on one or more lymph node areas other than the axilla to 297 patients. Although two of the 446 node-negative patients received adjuvant chemotherapy, none of them received adjuvant hormonal therapy. Of the 437 node-positive patients (for nine patients, nodal status was missing), adjuvant chemotherapy (mainly CMF; cyclophosphamide, methotrexate, 5-fluorouracil) was given to 146 patients, whereas 59 patients received hormonal therapy either alone (45 patients) or in combination with chemotherapy (14 patients). All patients were routinely examined every 3–6 months during the first 5 years of follow-up and once a year thereafter. During follow-up, 367 patients (41%) showed relapse and were counted as failures in the analysis for relapse-free survival. Fifty-seven patients (6%) died without evidence of disease and were censored at last follow-up in

**Table 1** Relationships between uPA and PAI-1 levels in 892 cytosols and pellet extracts with patient and tumour characteristics

| Characteristics | Number of patients[a] | Percentage of tumours above the median value[b] | | | |
|---|---|---|---|---|---|
| | | $uPA_{cyt.}$[c] | $uPA_{pel.}$[c] | $PAI\text{-}1_{cyt.}$[c] | $PAI\text{-}1_{pel.}$[c] |
| All patients | 892 | 50 | 50 | 50 | 50 |
| Age at surgery (years) | | | | | |
| ≤40 | 122 | 53 | 50 | 55 | 52 |
| 41–55 | 308 | 49 | 47 | 46 | 43 |
| 56–70 | 295 | 53 | 55 | 51 | 58 |
| > 70 | 167 | 46 | 47 | 53 | 48 |
| *P*-value[d] | | 0.12 | 0.97 | 0.32 | 0.24 |
| Menopausal status | | | | | |
| Premenopausal | 368 | 51 | 47 | 47 | 44 |
| Post-menopausal | 524 | 50 | 53 | 52 | 54 |
| *P*-value[e] | | 0.32 | 0.51 | 0.07 | 0.005 |
| Tumour size | | | | | |
| $T_1$ (≤ 2 cm) | 407 | 48 | 57 | 46 | 48 |
| $T_2$ (> 2–5 cm) | 399 | 52 | 45 | 52 | 52 |
| $T_{3/4}$ (> 5 cm) | 86 | 51 | 42 | 57 | 46 |
| *P*-value[f] | | 0.32 | <0.001 | 0.03 | 0.74 |
| Nodal status | | | | | |
| $N_0$ | 446 | 48 | 52 | 47 | 51 |
| $N_{1–3}$ | 216 | 53 | 53 | 52 | 50 |
| $N_{>3}$ | 221 | 52 | 44 | 55 | 48 |
| *P*-value[f] | | 0.44 | 0.05 | 0.30 | 0.55 |
| Grade | | | | | |
| Well/moderate | 152 | 51 | 50 | 41 | 46 |
| Poor | 510 | 51 | 51 | 54 | 53 |
| *P*-value[e] | | 0.60 | 0.83 | 0.006 | 0.04 |
| ER positive[g] | | | | | |
| No | 209 | 65 | 57 | 64 | 57 |
| Yes | 682 | 46 | 48 | 46 | 48 |
| *P*-value[d] | | 0.001 | 0.003 | 0.08 | <0.001 |
| PgR positive[g] | | | | | |
| No | 249 | 61 | 55 | 63 | 61 |
| Yes | 628 | 46 | 48 | 45 | 45 |
| *P*-value[d] | | 0.08 | 0.33 | <0.001 | <0.001 |

[a]Owing to missing values the numbers do not always add up to 892. [b]The median values in ng mg$^{-1}$ protein were 0.73 for $uPA_{cyt.}$, 7.26 for $uPA_{pel.}$, 1.62 for $PAI\text{-}1_{cyt.}$ and 5.29 for $PAI\text{-}1_{pel.}$ [c]$uPA_{cyt.}$, $uPA_{pel.}$, $PAI\text{-}1_{cyt.}$ and $PAI\text{-}1_{pel.}$ denote uPA and PAI-1 levels determined with the in-house assays in cytosol (cyt.) and pellet extracts (pel.) respectively. [d]*P*-value for Spearman rank correlation. [e]*P*-value for Wilcoxon rank sum test (for grade: well and moderate combined). [f]*P*-value for Kruskal–Wallis test, including a Wilcoxon-type test for trend. [g]Cut-off point used for ER and PgR, 10 fmol mg$^{-1}$ protein.

the analysis for relapse-free survival. Two hundred and twenty-eight patients (26%) died after a previous relapse. A total of 285 (63 + 222) patients (32%) were counted as failures in the analysis for overall survival. The median follow-up period of patients alive was 99 months (range 12–167 months). Further characteristics of patients and tumours are listed in Table 1.

### Tumour tissue extraction

Tumour tissues were stored in liquid nitrogen and pulverized in the frozen state with a microdismembrator as recommended by the European Organization for Research and Treatment of Cancer (EORTC, 1980) for processing of breast tumour tissue for cytosolic determination of steroid hormone receptors (ER and PgR) (EORTC Breast Cancer Cooperative Group). The resulting tissue powder was homogenized in EORTC receptor buffer [10 mM dipotassium hydrogen phosphate, containing 1.5 mM dipotassium chloride EDTA, 3 mM sodium azide, 10 mM monothioglycerol and 10% (v/v) glycerol, pH 7.4]. The homogenate was centrifuged for 30 min at 100 000 ***g*** and 4°C to obtain the supernatant fraction (cytosol). The 100 000 ***g*** pellets were rehomogenized with an Ultraturrax tissue homogenizer in 20 mM Tris-HCl (pH 8.5) containing 125 mM sodium chloride. After the addition of Triton X-100 to a final concentration of 1% and subsequent incubation for 16–20 h at 4°C, the supernatant fractions obtained by centrifugation at 30 000 ***g*** at 4°C were designated as pellet extracts.

### Steroid hormone receptor assays

ER and PgR levels were determined by ligand binding assay or enzyme immunoassay in cytosols as described earlier (Foekens et al, 1989). The cut-off level used to classify tumours as ER or PgR positive and negative was 10 fmol $mg^{-1}$ cytosolic protein.

### ELISAs for uPA and PAI-1

The antigen levels of uPA and PAI-1 were assessed with ELISAs. For determination of their levels in cytosols, two different assay formats were used, i.e. the previously evaluated ELISAs (Foekens et al, 1992, 1994), using reagents from kits which are now commercially available (American Diagnostica, Greenwich, CT, USA) and the newly established uPA and PAI-1 ELISAs based on polyclonal antibodies (Grebenschikov et al, 1997). The latter 'in-house' ELISAs were also used for the determination of uPA and PAI-1 levels in the corresponding pellet extracts. The respective antigen levels are referred to as $uPA_{in\text{-}house\ assay}$, $PAI\text{-}1_{in\text{-}house\ assay}$, $uPA_{AD}$ and $PAI\text{-}1_{AD}$ in the tables and figures. Triton X-100 did not have any influence on the ELISAs up to a concentration of 1%. An aliquot of a pooled breast tumour cytosol sample was analysed in each assay run to assess the between-assay variation, which was found to be below 15% for all assays used. The within-assay variation of samples measured in duplicate were all below 5%.

### Protein determinations

The Bradford method for protein analysis (Bradford, 1976) was used with the Bio-Rad reagent with human serum albumin (HSA) (KabiVitrum, Stockholm, Sweden) as a standard in order to express antigen levels per mg of total protein. Triton X-100 up to a concentration of 1% did not interfere with the protein determination in pellet extracts.

### Statistical analysis

The strength of the associations of uPA and PAI-1 levels determined in cytosols and pellet extracts with each other, age and steroid hormone receptor status was tested by Spearman rank correlation ($r_s$). The associations of uPA and PAI-1 with other clinical variables were tested with the non-parametric Wilcoxon rank-sum test or the Kruskal–Wallis test, including a Wilcoxon-type test for trends across ordered groups when appropriate. Relapse-free and overall survival probabilities were calculated by the actuarial method of Kaplan and Meier (1958). The log-rank test for trend was used to test ordered variables. Both uni- and multivariate analyses, including tests for interactions, were performed using the Cox proportional hazard model, and the associated likelihood ratio test was used to test for differences. To include the largest possible number of patients in multivariate analyses, patients with missing values for ER or PgR ($n$ = 16) and missing information on nodal status ($n$ = 9) were included as separate groups (not shown in the tables). All computations were carried out with the Stata statistical package, release 5.0 (Stata, College Station, TX, USA). Two-sided $P$-values below 0.05 were considered to be statistically significant.

**Table 2** Levels of uPA and PAI-1 in cytosols and pellet extracts

| Sample | First quartile | Median | Third quartile | Range | Mean ± s.d. |
|---|---|---|---|---|---|
| *Cytosols* | | | | | |
| $uPA_{AD}$ | 0.20 | 0.60 | 1.25 | 0.00–7.73 | 0.91 ± 0.98 |
| $PAI\text{-}1_{AD}$ | 8.97 | 15.43 | 25.48 | 0.02–479.38 | 22.23 ± 29.79 |
| $uPA_{in\text{-}house\ assay}$ | 0.38 | 0.73 | 1.24 | 0.02–12.66 | 0.95 ± 0.95 |
| $PAI\text{-}1_{in\text{-}house\ assay}$ | 1.06 | 1.62 | 2.62 | 0.14–48.62 | 2.43 ± 3.37 |
| *Pellet extracts* | | | | | |
| $uPA_{in\text{-}house\ assay}$ | 4.14 | 7.26 | 12.53 | 0.00–315.40 | 11.3–21.83 |
| $PAI\text{-}1_{in\text{-}house\ assay}$ | 3.29 | 5.29 | 8.64 | 0.26–300.60 | 9.3 ± 18.11 |

The antigen levels of uPA and PAI-1 were determined in cytosols and pellet extracts with the ELISAs using commercial reagents ($uPA_{AD}$ and $PAI\text{-}1_{AD}$) and the in-house-developed ELISAs ($uPA_{in\text{-}house\ assay}$ and $PAI\text{-}1_{in\text{-}house\ assay}$). The antigen levels are expressed as ng $mg^{-1}$ total protein.

© Cancer Research Campaign 1999

### RESULTS

### uPA and PAI-1 antigen determinations in cytosols and pellet extracts

The antigen levels of uPA and PAI-1 in 892 cytosols were determined with two different ELISA methods using commercial reagents and in-house-developed assays (Table 2). The mean and median levels of uPA as determined by both ELISAs were similar. However, the mean and median PAI-1 levels assessed by the two ELISAs differed by a factor of 10, the in-house ELISA measuring values ten times lower than the ELISA with commercial reagents. The overall association between the two ELISA methods is demonstrated by the highly significant correlations between the cytosolic levels of uPA ($r_s$ = 0.85, $P$ < 0.0001) and of PAI-1 ($r_s$ = 0.90, $P$ < 0.0001) (Figure 1A and B). When compared with the cytosolic levels of uPA and PAI-1 assessed with the in-house ELISAs, the mean and median levels determined in the pellet extracts were substantially higher, differing from one another by a factor of 10 (uPA) and a factor of 4 (PAI-1) respectively (Table 2). Clear and statistically significant correlations were found between the antigen levels in cytosols and corresponding pellet extracts ($r_s$ = 0.60, $P$ < 0.0001 for uPA and $r_s$ = 0.65, $P$ < 0.0001 for PAI-1) (Figure 1C and D). In addition, the levels of uPA and PAI-1 determined with the in-house assays significantly correlated with each other ($r_s$ = 0.66, $P$ < 0.0001 for cytosols and $r_s$ = 0.58, $P$ < 0.0001 for pellet extracts).

### Relation of uPA and PAI-1 levels to patient and tumour characteristics

The associations of cytosolic uPA and PAI-1 levels analysed with the commercial reagents with patient and tumour characteristics have been documented previously (Foekens et al, 1992, 1994). Therefore, in the present study, merely the levels of uPA and PAI-1 determined in cytosols and pellet extracts with the newly established in-house assays were related to patient and tumour characteristics (Table 1). The levels of uPA or PAI-1, when measured in cytosols, were not significantly related to age, menopausal status or nodal status. In contrast to the levels of uPA in cytosols, those of PAI-1 were higher in larger tumours. Similar results were obtained for the pellet extracts, with the exception of

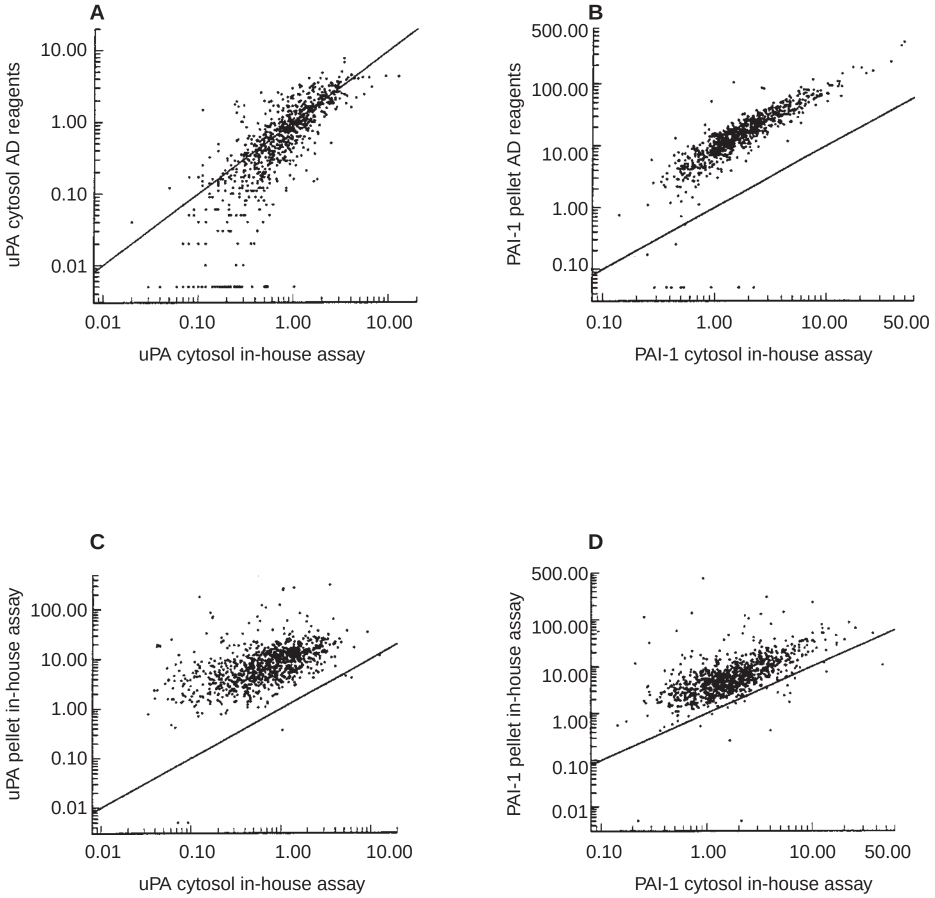

**Figure 1** Comparison of results obtained with different ELISAs for uPA and PAI-1. Antigen levels of uPA and PAI-1 in 892 cytosols were determined with two different ELISA methods using commercial (American Diagnostica, AD) reagents and in-house-developed assays. The scatter plots present the overall correlation between the results obtained with the ELISAs for uPA (**A**) and PAI-1 (**B**). The solid lines correspond to the lines of equality. Likewise, the antigen levels assessed in cytosols and pellet extracts with the in-house ELISA for uPA (**C**) and PAI-1 (**D**) were correlated with one another. Values below the assay sensitivity were set at half the value of the detection limits of each ELISA. The antigen levels were all expressed as ng mg$^{-1}$ of total protein measured in the cytosols and pellet extracts

*© Cancer Research Campaign 1999*

**Table 3** Cox univariate and multivariate analysis of relapse-free and overall survival

| Factor | Relapse-free survival | | | Overall survival | | |
|---|---|---|---|---|---|---|
| | Univariate *P*-value | Multivariate *P*-value | Relative relapse rate[a] | Univariate *P*-value | Multivariate *P*-value | Relative death rate[a] |
| Age and menopausal status | <0.001[b] | <0.001[b] | | <0.001[b] | <0.001[b] | |
| Age/premenopausal[c] | | | 0.63 (0.49–0.81) | | | 0.83 (0.59–1.17) |
| Age/postmenopausal[c] | | | 1.01 (0.86–1.18) | | | 1.38 (1.17–1.63) |
| Post- vs. premenopausal[d] | | | 1.40 (0.90–2.17) | | | 1.18 (0.68–2.06) |
| Tumour size | <0.001 | 0.06 | | <0.001 | 0.002 | |
| 2–5 cm vs. ≤ 2 cm | | | 1.21 (0.95–1.54) | | | 1.24 (0.93–1.64) |
| > 5 cm vs. ≤ 2 cm | | | 1.56 (1.07–2.27) | | | 2.02 (1.38–2.98) |
| Nodal status | <0.001 | <0.001 | | <0.001 | <0.001 | |
| $N_{1-3}$ vs. $N_0$ | | | 1.53 (1.11–2.10) | | | 2.11 (1.49–2.98) |
| $N_{>3}$ vs. $N_0$ | | | 2.99 (2.22–4.03) | | | 3.59 (2.58–4.99) |
| Adjuvant therapy (yes vs. no) | 0.06[e] | 0.01 | 0.68 (0.50–0.92) | <0.001[e] | 0.02 | 0.68 (0.49–0.95) |
| ER/PgR[f] | 0.001 | 0.05 | | <0.001 | <0.001 | |
| Pos/neg vs. neg/neg | | | 0.64 (0.42–0.95) | | | 0.54 (0.35–0.82) |
| Neg/pos vs. neg/neg | | | 0.77 (0.49–1.22) | | | 0.52 (0.31–0.87) |
| Pos/pos vs. neg/neg | | | 0.70 (0.53–0.91) | | | 0.45 (0.33–0.59) |

[a]Relative hazard rate (95% confidence interval) of multivariate analysis. [b]Age and menopausal status combined. [c]Age in decades tested separately for pre- and post-menopausal patients. [d]Post-menopausal compared with premenopausal. [e]In node-positive patients. [f]Cut-off points used for ER and PgR, 10 fmol $mg^{-1}$ protein.

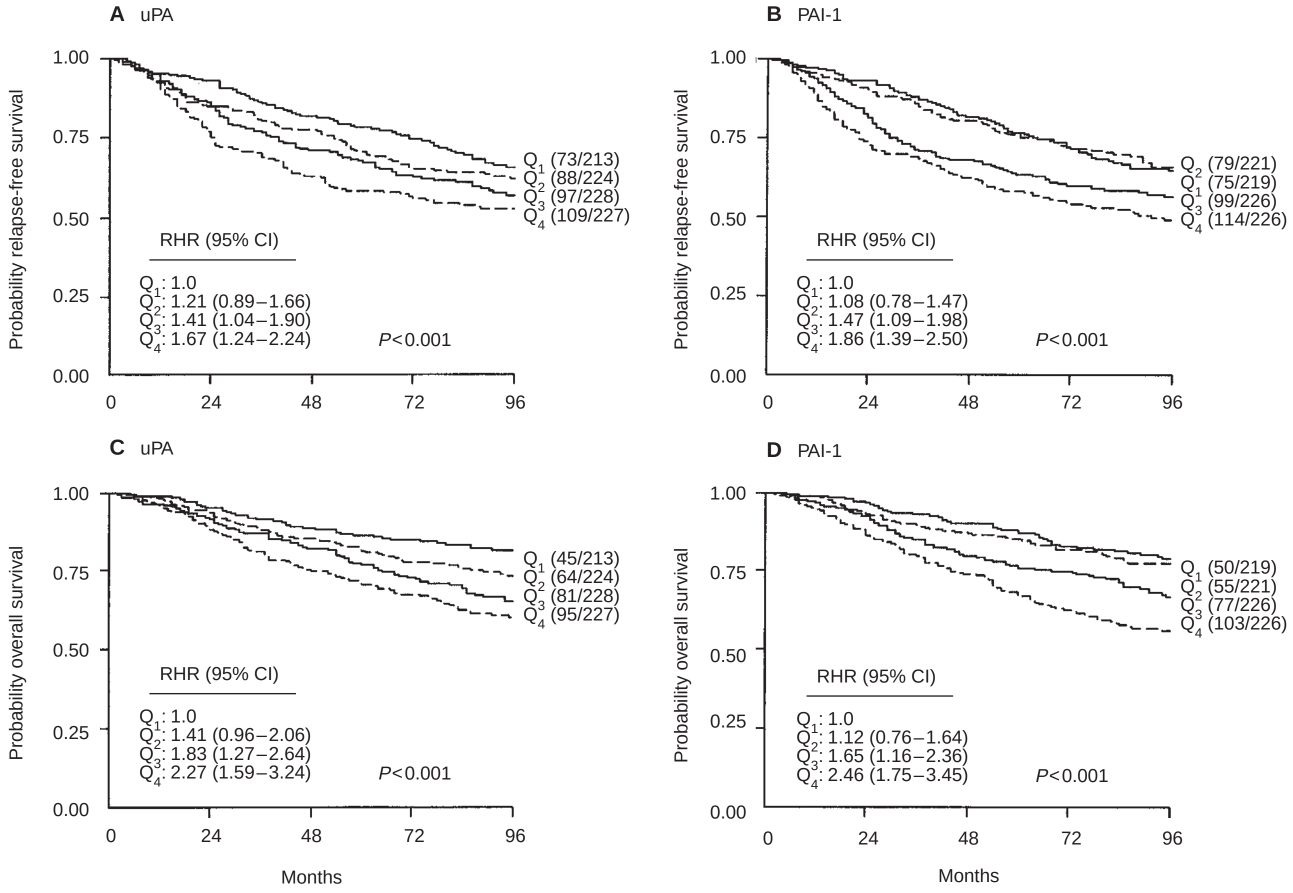

**Figure 2** Actuarial relapse-free and overall survival curves for all 892 breast cancer patients stratified by the levels of uPA (**A**) and (**C**) and PAI-1 (**B**) and (**D**) determined in cytosolic extracts of tumour tissue. Patients were divided into groups according to the quartiles ($Q_1$–$Q_4$) of the respective levels, as listed in Table 2. Shown are the *P*-values from the log-rank test for trend. RHR (95% CI), relative hazard rate (95% confidence interval) calculated by Cox multivariate regression analysis. In parentheses, the number of failures/total number of patients in each group

 © Cancer Research Campaign 1999

the observed negative association of uPA with tumour size and with nodal status and the higher PAI-1 levels in tumours from post-menopausal patients. Neither cytosolic uPA levels nor those measured in pellet extracts were associated with tumour grade. In contrast, the levels of PAI-1 were significantly higher in poorly differentiated tumours. In general, lower levels of uPA and PAI-1 were measured in steroid hormone receptor-positive tumours. However, these negative correlations were very weak and the strongest Spearman correlation coefficient observed was $r_s = -0.15$.

### Relation of uPA and PAI-1 levels to (relapse-free) survival

In univariate and multivariate Cox regression analyses, classical prognostic variables including age and menopausal status, tumour size, nodal status and steroid hormone receptor status were all significantly associated with the length of relapse-free survival (RFS) and overall survival (OS) (Table 3). Moreover, adjuvant treatment was associated with a favourable prognosis. In univariate analyses, poor differentiation grade of the tumour was associated with an early relapse ($P < 0.001$) and death ($P = 0.01$). Grade was not included in the basic multivariate model for RFS and OS shown in Table 3 because of too many missing values. To study their possible relationships with RFS and OS, the levels of uPA and PAI-1 measured with the in-house ELISAs were divided into four groups ($Q_1$–$Q_4$) by their respective quartiles, as shown in Table 2. There was a trend towards a worse RFS and OS with increasing cytosolic levels of uPA and PAI-1, the best prognosis being observed in patients with tumours containing uPA or PAI-1 levels in the first quarter ($Q_1$) and the worst prognosis being observed in patients with tumours containing uPA and PAI-1 levels in the fourth quarter ($Q_4$) (Figure 2). Similar relationships with poor RFS and OS, although less pronounced, were seen for uPA and PAI-1 levels measured in the pellet extracts (data not shown). In Cox multivariate regression analyses corrected for age, menopausal status, tumour size, lymph node status, steroid hormone receptor status and adjuvant therapy (basic multivariate model depicted in Table 3), cytosolic uPA and PAI-1 levels were significantly related to a poor RFS and OS, either when added as a categorical or as a log-transformed continuous variable. Comparable, although weaker, relationships with a poor RFS and OS were found for uPA and PAI-1 measured in the pellet extracts (Table 4). Regarding the prognostic value of uPA and PAI-1 as assessed with the commercial reagents, the levels of both components in cytosols, also divided into quarters, added significantly to the model, with relative rates of relapse and of death comparable to those found for the in-house assays (Table 4).

**Table 4** Cox multivariate analysis of relapse-free and overall survival

| Factor | Relapse-free survival | | Overall survival | |
|---|---|---|---|---|
| | Relative relapse rate[a] | *P*-value[b] | Relative relapse rate[a] | *P*-value[b] |
| *Cytosols*[c] | | | | |
| $uPA_{in\text{-}house\ assay}$ | | 0.02 | | <0.001 |
| $Q_2$ vs. $Q_1$ | 1.22 (0.89–1.67) | | 1.39 (0.95–2.05) | |
| $Q_3$ vs. $Q_1$ | 1.32 (0.97–1.79) | | 1.80 (1.24–2.60) | |
| $Q_4$ vs. $Q_1$ | 1.61 (1.19–2.18) | | 2.18 (1.51–3.13) | |
| $PAI\text{-}1_{in\text{-}house\ assay}$ | | <0.001 | | <0.001 |
| $Q_2$ vs. $Q_1$ | 1.21 (0.88–1.67) | | 1.28 (0.87–1.88) | |
| $Q_3$ vs. $Q_1$ | 1.47 (1.08–1.98) | | 1.59 (1.11–2.27) | |
| $Q_4$ vs. $Q_1$ | 1.88 (1.39–2.53) | | 2.31 (1.63–3.26) | |
| $uPA_{AD}$ | | 0.002 | | <0.001 |
| $Q_2$ vs. $Q_1$ | 1.52 (1.12–2.07) | | 1.88 (1.30–2.71) | |
| $Q_3$ vs. $Q_1$ | 1.23 (0.90–1.68) | | 1.67 (1.15–2.42) | |
| $Q_4$ vs. $Q_1$ | 1.73 (1.27–2.34) | | 2.35 (1.63–3.39) | |
| $PAI\text{-}1_{AD}$ | | <0.001 | | <0.001 |
| $Q_2$ vs. $Q_1$ | 1.14 (0.83–1.57) | | 1.21 (0.83–1.78) | |
| $Q_3$ vs. $Q_1$ | 1.56 (1.14–2.11) | | 1.63 (1.13–2.34) | |
| $Q_4$ vs. $Q_1$ | 1.84 (1.36–2.49) | | 2.41 (1.70–3.42) | |
| *Pellet extracts*[c] | | | | |
| $uPA_{in\text{-}house\ assay}$ | | 0.06 | | <0.001 |
| $Q_2$ vs. $Q_1$ | 1.03 (0.77–1.39) | | 1.40 (0.98–2.01) | |
| $Q_3$ vs. $Q_1$ | 1.05 (0.77–1.42) | | 1.72 (1.19–2.47) | |
| $Q_4$ vs. $Q_1$ | 1.43 (1.07–1.91) | | 2.08 (1.46–2.95) | |
| $PAI\text{-}1_{in\text{-}house\ assay}$ | | 0.003 | | <0.001 |
| $Q_2$ vs. $Q_1$ | 1.10 (0.80–1.51) | | 1.12 (0.77–1.63) | |
| $Q_3$ vs. $Q_1$ | 1.44 (1.06–1.95) | | 1.61 (1.14–2.28) | |
| $Q_4$ vs. $Q_1$ | 1.67 (1.24–2.27) | | 2.11 (1.50–2.97) | |

[a]Relative hazard rate (95% confidence interval) of multivariate analysis corrected for the basic model given in Table 3, including age/menopausal status, tumour size, nodal status, ER/PgR status and adjuvant therapy. Factors were added separately to the basic model. [b]*P*-values for the associated likelihood ratio test for the quarters. [c]$Q_1$, first quarter; $Q_2$, second quarter; $Q_3$, third quarter; $Q_4$, fourth quarter. The quartiles of the antigen levels determined with the in-house assays ($uPA_{in\text{-}house\ assay}$ and $PAI\text{-}1_{in\text{-}house\ assay}$) and the commercial reagents ($uPA_{AD}$ and $PAI\text{-}1_{AD}$) are listed in Table 2.

© Cancer Research Campaign 1999

There were no statistically significant interactions of uPA or PAI-1 with each other or with any of the classical prognostic parameters in the analyses for RFS and OS. Therefore, there are no reasons to assume that the prognostic value of uPA and/or PAI-1 is different in subgroups of patients. To visualize the combined prognostic value of uPA and PAI-1 with respect to the application of adjuvant therapy in the clinically important subgroup of node-negative patients, both factors were divided by their median value. The resulting actuarial RFS and OS curves as a function of combined uPA and PAI-1 status as determined by the in-house ELISAs are shown in Figure 3.

### DISCUSSION

In the present study, we evaluated the clinical relevance of uPA and PAI-1 antigen levels as measured in 892 breast tumour cytosols and the corresponding 100 000 ***g*** pellets re-extracted with detergent (Triton X-100)-containing buffer using newly developed ELISAs for the respective components. In this relatively large series of patients, the prognostic value of uPA and PAI-1 in breast carcinomas documented in previous prospective and retrospective studies as reviewed by Duffy (1996) and Schmitt et al (1997) has been confirmed. With respect to the analysis of cytosols, high levels of uPA and PAI-1 were associated with a significantly shorter RFS and OS. Moreover, in multivariate analyses which included classical prognostic parameters, cytosolic levels of both uPA and PAI-1 appeared to be important independent predictors of early relapse and death. The prognostic significance was ascertained by comparing RFS and OS for patients divided into quarters of the corresponding uPA and PAI-1 antigen levels, and by analysing RFS and OS as a function of uPA and PAI-1 analysed as continuous variables. The evaluation of survival by means of the quarters of antigen levels to discriminate between low-risk and high-risk patients represents a more unprejudiced approach compared with determination of optimized cut-off points which results in overfitting.

Interestingly, the 100 000 ***g*** pellets, which may be considered as incidental products of ultracentrifugation when preparing the cytosolic fractions, also provide prognostic information. However, when using the same assay, the pellet extracts contained substantially higher concentrations of uPA and PAI-1 (Table 2), but provided less prognostic information than the cytosolic extracts (Table 4). These findings seem to be in apparent contrast to those obtained by Jänicke et al (1994*a*), who demonstrated that determination of uPA, but not PAI-1, in detergent extracts of breast tumour tissue yields improved prognostic information (expressed as relative risk to relapse) compared with uPA in cytosol fractions. In the last investigation, the detergent was applied directly to tumour homogenates, whereas in the present study pellets which remained after preparation of the cytosols were treated with detergent.

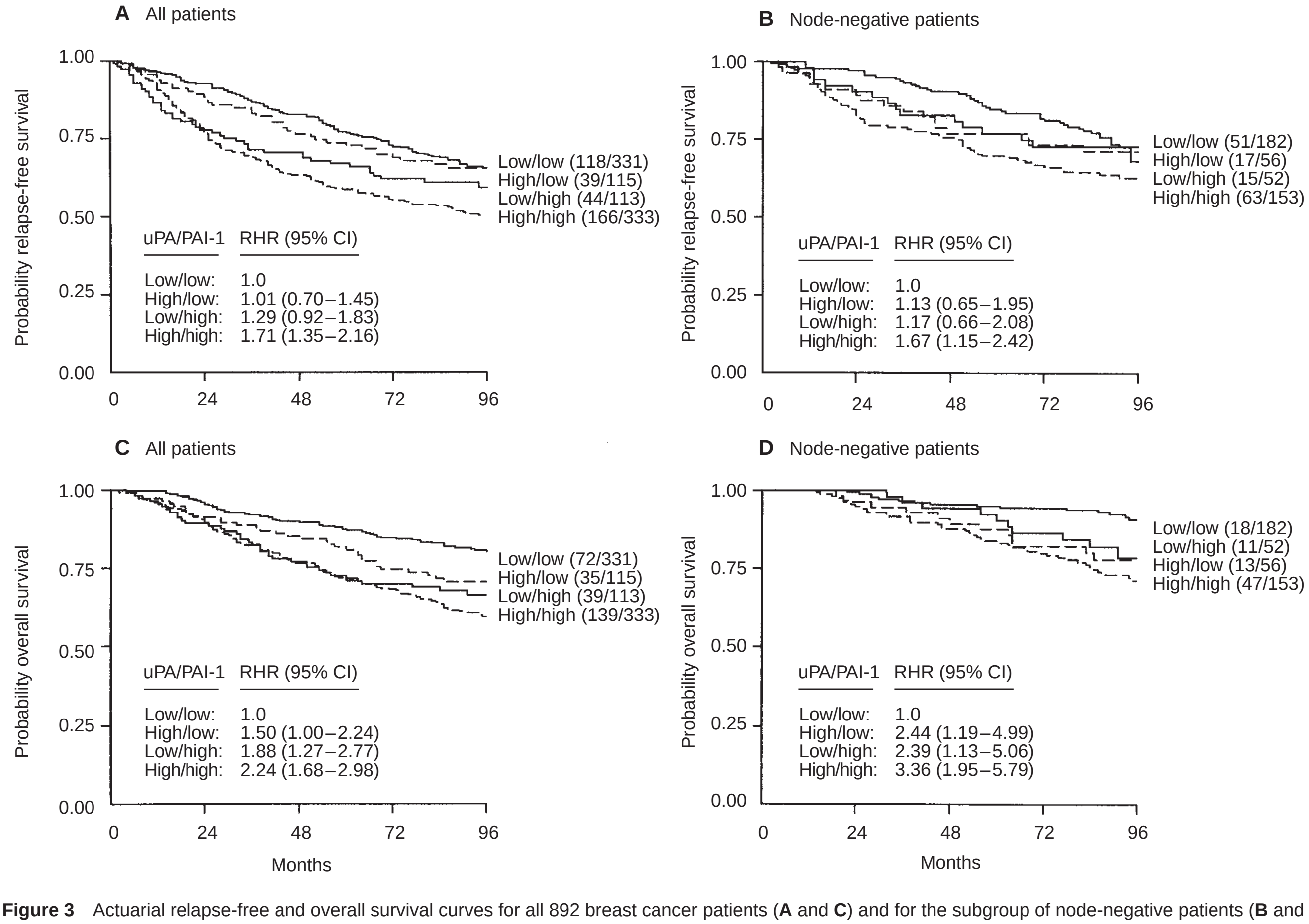

**Figure 3** Actuarial relapse-free and overall survival curves for all 892 breast cancer patients (**A** and **C**) and for the subgroup of node-negative patients (**B** and **D**) stratified by the combined levels of uPA and PAI-1 determined in cytosolic extracts of tumour tissue. Patients were divided into groups with levels below ('low') and above ('high') the median value of the respective uPA and PAI-1 levels, as listed in Table 2. RHR (95% CI), relative hazard rate (95% confidence interval) calculated by Cox multivariate regression analysis. In parentheses, the number of failures/total number of patients in each group

© Cancer Research Campaign 1999

Although the detergent-treated homogenates, as used by Jänicke et al (1994*a*), would contain uPA released from membranous components (uPAR) besides cytosolic uPA, our pellet extracts should contain exclusively the membrane-associated form of uPA. The possible additional prognostic value of uPA/uPAR complexes remains to be proven, for which purpose a recently developed ELISA for measurement of these complexes (De Witte et al, 1997) can be used.

In the clinical studies referred to above, various ELISAs have been used for the quantitative determination of uPA and PAI-1 in extracts of breast tumour biopsies. These ELISAs, whether applied as commercial kits or as in-house immunoassays, are all based on different combinations of monoclonal and/or polyclonal antibodies which are specifically directed against the component to be assessed in the biological sample. The newly developed in-house ELISAs for uPA and PAI-1 consist of specific polyclonal antibodies for catching and detection of analyte (Grebenschikov et al, 1997). In contrast, in the uPA and PAI-1 ELISA format composed of commercial reagents, a combination of monoclonal and polyclonal antibodies (PAI-1 ELISA) or only monoclonal antibodies (uPA ELISA) were used. The use of different antibodies with different specificities and affinities, as well as the heterogeneity of uPA in tumour samples, most probably underlies the disperse distribution of data when examining the overall association between the ELISA methods for uPA (Figure 1A). Indeed, uPA can be present in several different forms in tumour tissue extracts, including single-chain pro-uPA, two-chain uPA, amino-terminal fragment (ATF) of uPA, complexes of (pro-) uPA with uPAR, PAI-1 and/or PAI-2, and different ELISA formats may recognize each of the various forms of uPA with different efficiencies. Moreover, the combined commercial reagents in the ELISA format for uPA do not detect LMW-uPA (Grebenschikov et al, 1997). Notwithstanding the variation between the values obtained by the two uPA ELISAs, the mean and median levels of uPA determined by both methods were highly comparable to each other. The scatter plot for the overall association between the PAI-1 data reveals a very limited spread of data, despite a systematic bias relative to the line of equality over the whole range of PAI-1 concentrations (Figure 1B). This difference (approximately a factor of 10) between PAI-1 antigen levels obtained by both ELISAs may be explained, for the largest part, by a difference in calibration of the PAI-1 standards used in the two ELISAs. In fact, both PAI-1 ELISAs used standards with different immunoreactive potencies, as reflected by the divergent ELISA responses obtained when both standards were assayed in the in-house ELISA for PAI-1 (Grebenschikov et al, 1997). Moreover, when using the commercial PAI-1 standard in the two assay formats, the PAI-1 antigen values measured in cytosols by both ELISA methods were almost equal to one another, as reported earlier (Grebenschikov et al, 1997). Furthermore, the narrow dispersion of data is indicative of assessment of PAI-1, which is considered to be present in just a few different forms, with comparable efficiencies in both ELISA methods.

A direct comparison of both uPA and PAI-1 levels measured in breast tumour extracts, including cytosols, with those reported in the literature is quite difficult given the lack of standardization (Benraad et al, 1996) because different antibodies and standards as well as different extraction buffers were used in the various studies. The application of different ELISA methods, each using different antibodies with different specificities towards the various components, may disclose which of the molecular forms of uPA and/or PAI-1 present in tumour tissue extracts is responsible for the observed relationship with prognosis. To specifically address this aspect, an alternative study design is desired in which identical aliquots of the patient samples are simultaneously analysed by the different assays. The strong statistically significant correlations between the cytosolic levels of either uPA or PAI-1, as determined by the different ELISA formats in this investigation, suggest, however, that both methods exert comparable prognostic information, and that either assay can be used to assess the uPA and PAI-1 status of the breast tumour.

The corroboration of the prediction of prognosis on the basis of uPA and PAI-1, assessed by the independent ELISA methods for each of the components, once again emphasizes the established prognostic role and usefulness of these parameters in selection of primary breast cancer patients at high or low risk of recurrence. This also applies to the patients with node-negative disease, who as a group have a relatively favourable prognosis. Simultaneous measurement of uPA and PAI-1 might be helpful in identifying those node-negative patients who will experience a recurrence and might benefit from adjuvant therapy. Within this framework, the results of an ongoing German multicentre clinical study on adjuvant chemotherapy in node-negative breast cancer, based on selection by the tumour levels of uPA and PAI-1 (Jänicke et al, 1994*b*), are of special interest.

### REFERENCES

Andreasen PA, Georg B, Lund LR, Riccio A and Stacey SN (1990) Plasminogen activator inhibitors: hormonally regulated serpins. *Mol Cell Endocrinol* **68**: 1–19

Andreasen PA, Kjøller L, Christensen L and Duffy MJ (1997) The urokinase-type plasminogen activator system in cancer metastasis: a review. *Int J Cancer* **72**: 1–22

Benraad ThJ, Geurts-Moespot J, Grøndahl-Hansen J, Schmitt M, Heuvel JJTM, de Witte JH, Foekens JA, Leake RE, Brünner N and Sweep CGJ (1996) Immunoassays (ELISA) of urokinase-type plasminogen activator (uPA): report of an EORTC/BIOMED-1 workshop. *Eur J Cancer* **32A**: 1371–1381

Bradford MM (1976) A rapid and sensitive method for the quantitation of microgram quantities of protein utilizing the principle of protein–dye binding. *Anal Biochem* **72**: 248–254

Chapman HA (1997) Plasminogen activators, integrins, and the coordinated regulation of cell adhesion and migration. *Curr Opin Cell Biol* **9**: 714–724

De Witte H, Pappot H, Brünner N, Grøndahl-Hansen J, Høyer-Hansen G, Behrendt N, Guldhammer-Skov B, Sweep CGJ, Benraad ThJ and Danø K (1997) ELISA for complexes between urokinase-type plasminogen activator and its receptor in lung cancer tissue extracts. *Int J Cancer* **72**: 416–423

Danø K, Andreasen PA, Grøndahl-Hansen J, Kristensen P, Nielsen LS and Skriver L (1985) Plasminogen activators, tissue degradation, and cancer. *Adv Cancer Res* **44**: 139–266

Danø K, Behrendt N, Brünner N, Ellis V, Ploug M and Pyke C (1994) The urokinase receptor: protein structure and role in plasminogen activation and cancer invasion. *Fibrinolysis* **8**(suppl. 1): 189–203

Duffy MJ (1992) The role of proteolytic enzymes in cancer invasion and metastasis. *Clin Exp Metastasis* **10**: 145–155

Duffy MJ (1996) Proteases as prognostic markers in cancer. *Clin Cancer Res* **2**: 613–618

Duffy MJ, Reilly D, O'Sullivan C, O'Higgins N, Fennelly JJ and Andreasen PA (1990) Urokinase plasminogen activator, a new and independent prognostic marker in breast cancer. *Cancer Res* **50**: 6827–6929

EORTC Breast Cancer Cooperative Group (1980) Revision of the standards for the assessment of hormone receptors in human breast cancer; report of the second EORTC workshop, held on 16–17 March 1979, in The Netherlands Cancer Institute. *Eur J Cancer* **16**: 1513–1515

Fernö M, Bendahl P-O, Borg Å, Brundell J, Hirschberg L, Olsson H and Killander D (1996) Urokinase plasminogen activator, a strong independent prognostic factor in breast cancer, analysed in steroid receptor cytosols with a luminometric immunoassay. *Eur J Cancer* **32A**: 793–801

Foekens JA, Portengen H, van Putten WLJ, Peters HA, Krijnen HLJM, Alexieva-Figusch J and Klijn JGM (1989) Prognostic value of estrogen and progesterone

© Cancer Research Campaign 1999

receptors measured by enzyme immunoassays in human breast tumor cytosols. *Cancer Res* **49**: 5823–5228

Foekens JA, Schmitt M, van Putten WLJ, Peters HA, Bontenbal M, Jänicke F and Klijn JGM (1992) Prognostic value of urokinase-type plasminogen activator in 671 primary breast cancer patients. *Cancer Res* **52**: 6101–6105

Foekens JA, Schmitt M, van Putten WLJ, Peters HA, Kramer MD, Jänicke F and Klijn JGM (1994) Plasminogen activator inhibitor-1 and prognosis in primary breast cancer. *J Clin Oncol* **12**: 6148–1658

Grebenschikov N. Geurts-Moespot A, De Witte H, Heuvel J, Leake R, Sweep F and Benraad T (1997) A sensitive and robust assay for urokinase and tissue-type plasminogen activators (uPA and tPA) and their inhibitor type I (PAI-1) in breast tumor cytosols. *Int J Biol Markers* **12**: 6–14

Grøndahl-Hansen J, Christensen IJ, Rosenquist C, Brünner N, Mouridsen HT, Danø K and Blichert-Toft M (1993) High levels of urokinase-type plasminogen activator and its inhibitor PAI-1 in cytosolic extracts of breast carcinomas are associated with poor prognosis. *Cancer Res* **53**: 2513–2521

Jänicke F, Schmitt M, Ulm K, Gössner W and Graeff H (1989) Urokinase-type plasminogen activator antigen and early relapse in breast cancer. *Lancet* 8670: 1049

Jänicke F, Schmitt M, Hafter R, Hollrieder A, Babic R, Ulm K, Gössner W and Graeff H (1990) Urokinase-type plasminogen activator (u-PA) antigen is a predictor of early relapse in breast cancer. *Fibrinolysis* **4**: 69–78

Jänicke F, Schmitt M and Graeff H (1991) Clinical relevance of the urokinase-type and tissue-type plasminogen activators and of their type-1 inhibitor in breast cancer. *Semin Thromb Hemostas* **17**: 303–312

Jänicke F, Schmitt M, Pache L, Ulm K, Harbeck N, Höfler H and Graeff H (1993) Urokinase (uPA) and its inhibitor PAI-1 are strong and independent prognostic factors in node-negative breast cancer. *Breast Cancer Res Treat* **24**: 195–208

Jänicke F, Pache L, Schmitt M, Ulm K, Thomssen C, Prechtl A and Graeff H (1994*a*) Both the cytosols and detergent extracts of breast cancer tissues are suited to evaluate the prognostic impact of the urokinase-type plasminogen activator and its inhibitor, plasminogen activator inhibitor type 1. *Cancer Res* **54**: 2527–2530

Jänicke F, Thomssen C, Pache L, Schmitt M and Graeff H (1994*b*) Urokinase (uPA) and PAI-1 as selection criteria for adjuvant chemotherapy in axillary node-negative breast cancer patients. In *Prospects in Diagnosis and Treatment of Breast Cancer*, Schmitt M, Graeff H and Kindermann G (eds), pp. 207–218. Elsevier Publishers: Amsterdam

Kaplan EL and Meier P (1958) Nonparametric estimation from incomplete observation. *J Am Stat Assoc* **53**: 457–481

Liotta LA, Thorgeirsson UP and Garbisa S (1982) Role of collagenases in tumor cell invasion, tissue degradation, and cancer. *Cancer Metastasis Rev* **1**: 277–297

Mignatti P and Rifkin DB (1993) Biology and biochemistry of proteinases in tumor invasion. *Physiol Rev* **73**: 161–195

Schmitt M, Harbeck N, Thomssen C, Wilhelm O, Magdolen V, Reuning U, Ulm K, Höfler H, Jänicke F and Graeff H (1997) Clinical impact of the plasminogen activation system in tumor invasion and metastasis: prognostic relevance and target for therapy. *Thromb Haemost* **78**: 285–296

Spyratos F, Martin P-M, Hacène K, Romain S, Andrieu C, Ferrero-Poüs M, Deytieux S, Le Doussal V, Tubiana-Hulin M and Brunet M (1992) Multiparametric prognostic evaluation of biological factors in primary breast cancer. *J Natl Cancer Inst* **84**: 1266–1272

*© Cancer Research Campaign 1999*
